# Age-Dependent Up-Regulation of HCN Channels in Spiral Ganglion Neurons Coincide With Hearing Loss in Mice

**DOI:** 10.3389/fnagi.2018.00353

**Published:** 2018-11-06

**Authors:** Haitao Shen, Weilin Liu, Qiaowei Geng, Hongchen Li, Mingshun Lu, Peng Liang, Bo Zhang, Ebenezer N. Yamoah, Ping Lv

**Affiliations:** ^1^Laboratory of Pathology, Hebei Medical University, Shijiazhuang, China; ^2^Division of Cardiovascular Medicine, Hebei Province Geriatric Hospital, Shijiazhuang, China; ^3^Department of Pharmacology, Hebei Medical University, Shijiazhuang, China; ^4^Department of Physiology, School of Medicine, University of Nevada, Reno, NV, United States

**Keywords:** spiral ganglion neuron, HCN channel, age-related hearing loss, apoptosis, apoptosis-inducing factor

## Abstract

Age-related hearing loss (AHL) is the most common sensory disorder in the elderly population, and the etiologies are diverse. To understand the underlying mechanisms of AHL, one strategy is to identify correlates of the disease for comprehensive evaluation of treatment approaches. Dysfunction and degeneration of spiral ganglion neurons (SGNs) are major contributors to AHL. Previously, we showed that one of the changes in the aging auditory system is SGN excitability increase in mice. Since hyperpolarization-activated cyclic nucleotide-gated (HCN) channels play important roles in determining neuronal excitability, we predicted that HCN channels in SGNs are involved in AHL. To investigate the contribution of HCN channels to AHL, we examined the expression and biophysical properties of HCN channels in SGNs from adult (2–3 months) and 11–12-month-old mice. We report a dramatic increase of HCN channel current (Ih) in SGNs in old mice (11–12 months old). The results matched well with increased expression of HCN1 and HCN2 subunits, suggesting that upregulation of HCN channels in SGNs is one of the important facets of the aging SGNs. Moreover, the activity of Ih produced a major impact on the firing properties of SGNs in older mice. The upregulation of Ih may contribute to AHL by regulating SGN excitability. We assessed whether increased SGNs excitability dovetail with neurodegeneration. Apoptosis-inducing factor (AIF)-mediated apoptosis in SGNs was observed in old mice and activation of HCN channels mediates AIF activation. Thus, these findings demonstrate stark correlation between age-dependent increased expression of HCN channels and Ih, and apoptosis in SGNs, which may contribute towards the varied mechanisms of AHL.

## Introduction

Age-related hearing loss (AHL), also known as presbycusis, is the most common sensory disorder in the elderly population, affecting about 40% of people over 65 years old (Gates and Mills, 2005). It is characterized by decreased hearing sensitivity, and decreased ability to understand speech in a noisy environment, as well as slowed central processing of acoustic stimuli, and impaired sound localization (Gates and Mills, 2005).

AHL is associated with an age-dependent loss of sensory hair cells, spiral ganglion neurons (SGNs), and stria vascularis cells in the inner ear (Gates and Mills, 2005; Yamasoba et al., 2007). Hair cells and SGNs do not regenerate in mammals, and loss of these long-lived cochlear cells leads to permanent hearing impairment. A long-held view has been that age-related loss of SGNs occurs as a consequence of hair cell loss, which suggests loss of hair cell is the main cause of AHL (Fritzsch et al., 1997; Takeno et al., 1998). However, recent evidence demonstrates that damage to or loss of SGNs can occur independent of hair cell death and may serve as one of the primary sites for the initiations of AHL (Ryals and Westbrook, 1988; White et al., 2000; Linthicum and Fayad, 2009).

SGNs are the primary afferent neurons of the auditory system and provide the vital link between hair cells and the central auditory nuclei. SGNs convey signals from hair cells to the brain in a manner that preserves the amplitude, frequency, and temporal features of sound information (Geisler, 1998; Taberner and Liberman, 2005; Meyer and Moser, 2010). This signal transmission is critical for normal auditory processing and sound localization. Thus, functional decline of SGN functions result in hearing impairment. Our previous study showed SGNs excitability was altered in old vs. young mice, suggesting that disordered auditory signal transmission caused by alterations of SGN function may contribute to AHL (Lv et al., 2010). The hyperpolarization-activated cyclic nucleotide-gated (HCN) channels play an important role in determining neuron excitability (Ludwig et al., 1998; Robinson and Siegelbaum, 2003). HCN channels form heterotetramers or homotetramers, composed of HCN1–4 subunits, and can be activated by membrane hyperpolarization and depolarization (Ludwig et al., 1998; Robinson and Siegelbaum, 2003). Thus, the biophysical properties of HCN channels contribute to resting membrane potential (RMP) and firing properties in many different types of excitable cells and neurons (Robinson and Siegelbaum, 2003; Biel et al., 2009). Upregulation or downregulation of HCN channels may underlie abnormal excitability and result in neurological disorders, including epilepsy and neuropathic pain (Bakondi et al., 2009; Lewis and Chetkovich, 2011; Weng et al., 2012). In the auditory system, HCN channels have been identified in SGNs; however, whether and how alterations of HCN channels contribute towards age-dependent changes are still unknown (Chen, 1997; Mo and Davis, 1997; Yi et al., 2010; Kim and Holt, 2013).

To investigate how HCN channels may affect SGNs function and contribute towards age-related functional changes and neurodegeneration associated with AHL, we examined the expression and biophysical properties of HCN channels in SGNs from young and old mice. For pragmatic reasons we used C57BL/6 mice, which are known to undergo accelerated AHL (Zheng et al., 1999; Liu S. et al., 2012), resulting from mutations in *cdh23* gene a component of the mechano-transducer apparatus, and age-related SGN loss (Schettino and Lauer, 2013). Our data indicate that HCN channel current (Ih) density increased significantly with increased expression of HCN1 and HCN2 in SGNs in old mice. In addition, HCN channels had a major impact on RMP and excitability of SGNs from old mice. Moreover, upregulation of HCN channels correlates with activation of apoptosis-inducing factor (AIF)-mediated SGNs apoptosis in old mice. Collectively, our findings demonstrate that HCN channels play an important role in regulating SGN function, and alteration of HCN channels in SGNs may be involved in AHL.

## Materials and Methods

### Animals

This study was carried out in accordance with the recommendations of the Animal Care and Ethical Committee of Hebei Medical University. The protocol was approved by the Animal Care and Ethical Committee of Hebei Medical University (2016HBMU-0121065). All the C57BL/6 mice were bred in-house under a 12:12 h light-dark cycle. Mice were divided into young (2–3 months old) and old (~11–12 months old) groups.

### Auditory Brainstem Responses (ABR) Testing

Animals were anesthetized with an intraperitoneal injection of 100 mg/kg ketamine and 10 mg/kg xylazine. Platinum needle electrodes were placed subcutaneously at the vertex (reference electrode), behind the right ear (active electrode) and in the back (ground electrode). Auditory brainstem responses (ABRs) were measured in response to tone pips of 8, 12, 16, 20, 24, 28 and 32 kHz. ABR recordings were performed with a Tucker Davis Technologies (TDT) System III workstation running in a BioSigRP sound booth (IAC). The hearing threshold was defined as the lowest intensity to generate a reproducible ABR waveform.

### SGNs Morphometry and Counting

Paraffin-embedded cochlea specimens were sliced at 5 μm, stained with hematoxylin and eosin, and observed under a light microscope. The Rosenthal’s canal was divided into three regions: apex, middle and base. SGNs from these three regions of the cochlea were used for evaluation of morphometry and cell-counting (high-magnification, Olympus). We counted the cells in one field (apex, middle or base) in each section, and six representative sections were analyzed in one cochlea per mouse. In each group, 5–6 mice were used for SGN-counting.

### SGNs Culture

Isolation of SGNs followed a detailed procedure outlined in a previous study (Lv et al., 2010). Briefly, adult mice were killed and the temporal bones were removed in a solution containing MEM with Hank’s salt (Invitrogen) supplemented with 0.2 g/L kynurenic acid, 10 mM MgCl_2_, 2% fetal bovine serum (FBS; v/v), and 6 g/L glucose. The central spiral ganglion tissue was dissected out and split into apical and basal pieces across the modiolar axis. The tissue was digested in an enzyme mixture containing collagenase type I (1 mg/ml) and DNase (1 mg/ml) at 37°C for 15 min. After gentle trituration and centrifugation at 2,000 rpm for 5 min in 0.45 M sucrose, the cell pellets were reconstituted in 0.9 ml of culture media (Neurobasal™ A, supplemented with 2% B27 (v/v), 0.5 mM L-glutamine, 100 units/ml penicillin; Invitrogen). The freshly isolated SGNs were filtered through a 40-μm cell strainer and plated onto glass coverslips, pretreated with 0.5 mg/ml poly-D-lysine (Sigma-Aldrich) and 1 mg/ml laminin (Sigma-Aldrich). SGNs were kept in culture for 24–48 h before electrophysiological recordings.

### Electrophysiology

The whole-cell voltage-clamp technique was used in recording Ih from SGNs cell bodies. Fire-polished electrodes (3–4 MΩ) were pulled from borosilicate glass. The internal solution contained (in mM): KCl 112, MgCl_2_ 2, CaCl_2_ 0.1, HEPES 10, EGTA 1, K_2_ATP 5, pH 7.2 with KOH. The external solution contained (in mM): NaCl 125, KCl 5, MgCl_2_ 1, TEA-Cl 20, HEPES 10, glucose 5, CdCl_2_ 0.2, BaCl_2_ 0.1, TTX 0.001, 4-AP 1, pH 7.3 with NaOH. Ih was generated from a holding potential of −60 mV to potentials between −120 mV and −50 mV in 10-mV increment. The capacitative transients were used to estimate cell capacitance as an indirect measure of cell size. The mean values for SGN capacitance in young (2–3 month) mice was 23 ± 5 pF (*n* = 28) and old (11–12 month) mice was 26 ± 7 pF (*n* = 22), *p* = 0.73. To avoid potential caveat resulting from current rundown, only cells with stable current amplitude +5% change in total current amplitude, were analyzed and included in the report.

Whole-cell current-clamp recordings were performed, using bath solution containing (in mM): NaCl 130, KCl 5, MgCl_2_ 1, CaCl_2_ 1, HEPES 10, Glucose 10, pH 7.3 with NaOH. The internal pipette solution contained (in mM) KCl 112, MgCl_2_ 1, CaCl_2_ 0.01, K_2_ATP, HEPES 10, EGTA 0.5, pH 7.3 with KOH. The stock solutions of all channel blockers used were made either in double distilled water or DMSO and stored at −20°C. The final concentration of DMSO in the recording bath solution was ~0.001%. ZD7288 was purchased from Tocris Bioscience, and all other chemicals were purchased from Sigma. Liquid junction potentials (LJPs) were measured and corrected as described (Neher, 1992). LJPs were less than 2.5 mV and corrected, online. The amplifier build-in bridge balance was used to zero potential offsets and fluctuations before obtaining electrical assess to the cell.

Electrophysiological experiments were conducted using an Axopatch 200B amplifier (Molecular Devices, San Jose, CA, USA). Signals were filtered at 2 kHz with a low pass Bessel filter and digitizer at ≥20 kHz using a 12-bit acquisition system, Digidata 1332 (Axon Instruments), and pClamp 9.0 software (Molecular Devices, San Jose, CA, USA).

### RT-PCR

Cochleae were dissected from the temporal bone and the spiral ligament was removed (five mice per sample, six samples per group). Total RNA was extracted from the modiolus using RNAeasy kit (Qiagen, Hilden, Germany) according to the manufacturer’s instructions. The total RNA mixture was reverse transcribed to cDNA in a 25 μl reaction mixture containing 2 μg total RNA, 0.5 μl Oligo DT (0.5 μg/ml), 0.5 μl RNase inhibitor (30 U/μl), 2.5 μl dNTPs (10 mM), 5× real-time (RT) buffer, and 0.4 μl AMV (5 U/μl, Promega, Beijing, China). Transcript levels of HCN1–4 were evaluated by RT PCR, performed using a SYBR@Green Kit (Invitrogen) according to the manufacturer’s instructions. The sequences of primers used were as follows: glyceraldehyde-3-phosphate dehydrogenase (GAPDH), forward: 5′-ACCACAGTCCATGCCATCAC-3′, reverse: 5′-TCCACCACCCTGTTGCTGTA-3′, HCN1 forward: 5′-ACATGCTGTGCATTGGTTATGGCG-3′, reverse: 5′-AACAAACATTGCGTAGCAGGTGGC-3′, forward: 5′-ACTTCCGCACCGGCATTGTTATTG-3′, reverse: 5′-TCGATTCCCTTCTCCACTATGAGG-3′, HCN3 forward: 5′-CCTCATCCGCTACATACACCAGT-3′, reverse: 5′-GACACAGCAGCAACATCATTCC-3′, and HCN4 forward: 5′-GCATGATGCTTCTGCTGTGTCACT-3′, reverse: 5′-TTCACCATGCCATTGATGGACACC-3′. Gene expression was normalized against the housekeeping gene GAPDH. Relative gene expression was calculated by the comparative ΔΔCt method according to the manufacturer’s instructions.

### Western Blot

Total protein extracts were prepared from five mice modioli of each sample with RIPA buffer (25 mM Tris HCl, 150 mM NaCl, 1% NP-40, 1% sodium deoxycholate, 1% sodium dodecyl sulfate [SDS] and protease inhibitor cocktail). The homogenate was centrifuged at 14,000 *g* for 15 min at 4°C, and protein concentration in the supernatant was determined using a BCA protein assay. Equal amounts of protein were resolved by SDS-PAGE, and transferred to a nitrocellulose membrane. Membranes were blocked with 5% dry milk in PBS, probed with one of the primary antibodies (anti-HCN1, anti-HCN2, anti-HCN3 and anti-HCN4, 1:200; Alomone Labs), followed by the application of appropriate HRP-conjugated secondary antibodies as per the manufacturer’s recommendations. All western blots were visualized using an enhanced chemiluminescence system (Las3000, Fujifilm, Tokyo, Japan).

### Immunofluorescence

Cochleae were dissected from the temporal bone and fixed at 4°C in 4% paraformaldehyde in PBS overnight, processed sequentially with 10% EDTA, 10 and 30% sucrose at 4°C overnight, then embedded in optimal cutting temperature (OCT) for cryosectioning in the modiolar plane. Sections of 10 μm were washed in PBS, permeabilized in 0.1% Triton X-100 for 25 min, and then incubated for 60 min in a blocking solution containing 1% bovine serum albumin and 10% goat serum. The sections were incubated with primary antibody overnight at 4°C. The rinsed sections were then incubated (2 h; room temperature) in a fluorescent dye-conjugated secondary antibody. The following primary antibodies were used: rabbit anti-HCN1, anti-HCN2, anti-HCN3, anti-HCN4 (Alomone Labs), mouse anti-AIF (Gene Tex), rabbit anti cleaved caspase-3 (Cell Signaling Technology) and mouse anti neuronal class III ß-Tubulin (TUJ1; Covance). Secondary antibodies were fluorescein (FITC)-conjugated affinity purified goat anti rabbit IgG and Cy3-conjugated affinity purified goat anti mouse IgG (Jackson Labs). Images were captured with a Leica TCS SP5 confocal microscope.

SGNs were isolated from the mouse inner ear (five mice) and cultured for 72 h and incubated with forskolin (FSK; 20 μM) for 24 h. Neurons were fixed for 30 min with 4% paraformaldehyde in PBS, washed, and then permeabilized in 0.5% Triton X-100 in PBS for 5 min. The samples were incubated for 1 h in a blocking solution containing 1% bovine serum albumin in PBS, followed by 0.5% Triton X-1 with AIF antibody (Gene Tex) at 1:100. To identify neurons, samples were stained with an antibody against the neuronal marker TUJ1. Cells were then incubated with appropriate secondary antibodies overnight, washed, mounted using antifade mounting medium and viewed with a Leica TCS SP5 confocal microscope.

### Data Analysis

Results are expressed as the mean ± SEM. Statistical analysis was performed by ANOVA using the PRISM software, version 3.0 followed by either Tukey’s test for multiple comparisons or Student *t*-test. *P* < 0.05 was considered statistically significant. The number of animals and cells used are indicated in figure legends. Current magnitudes were measured using averages of the steady-state levels or peak detection routine in the pClamp software.

## Results

### HCN Channel Expression Is Increased in SGNs From Old Mice

**Figure 1:**
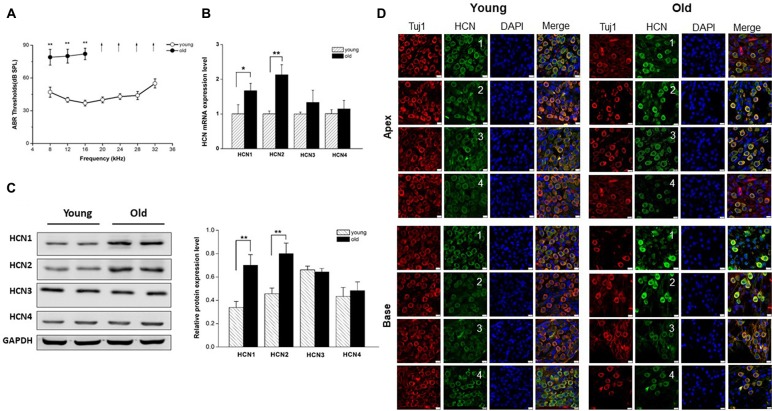
Increased expression of hyperpolarization-activated cyclic nucleotide-gated 1 (HCN1) and HCN2 in spiral ganglion neurons (SGNs) of old mice. **(A)** Auditory brainstem response (ABR) hearing thresholds were measured from young and old mice (*n* = 8). Arrows indicate that thresholds exceeded the upper limits of the tucker davis technologies (TDT) ABR system. **(B)** Real-time PCR of HCN subunits in SGNs from young and old mice, showed a significant increase of HCN1 and HCN2 at the mRNA level in old mice (*n* = 6). **(C)** Western blotting showed HCN1 and HCN2 expression level was significantly increased in the SGNs of old mice, but HCN3 and HCN4 were unchanged (*n* = 4). Data are means ± SEM, *p < 0.05, **p < 0.01. **(D)** Immunofluorescence showed that all of four HCN subunits (green) were present in both young and old SGNs. SGNs were labeled with the neuronal marker Tuj1 (red). The nuclei (blue) were stained with DAPI. Scale bar: 10 μm.

We first examined the auditory function in young and old mice by measuring ABR, an objective electrophysiological test of hearing function. As shown in Figure 1A, ABR thresholds from old mice were significantly elevated compared to those from young mice at low frequencies, and exceeded the upper limits of the ABR system at the middle and high frequencies, suggesting that auditory function declined in old mice.

It has been shown that membrane excitability changes occur in SGNs from old mice (Lv et al., 2010). Due to an important role of HCN channels in the regulation of neuron excitability, we investigated whether HCN channel expression levels were altered in SGNs during AHL. We examined expression of all known HCN channel subunits (HCN1-HCN4) in SGNs from young and old mice. As shown in Figure 1D, HCN1–4 subunits were present in young and old SGNs. Only HCN1 and HCN2 were increased significantly in SGNs from old mice at the mRNA and protein expression levels (Figures 1B,C). By contrast, HCN3 and HCN4 expression were statistically unchanged between young and old mice. These results suggest that HCN1 and HCN2 may contribute to upregulation of HCN channels in SGNs from old mice.

### Increased Ih in SGNs From Old Mice

**Figure 2:**
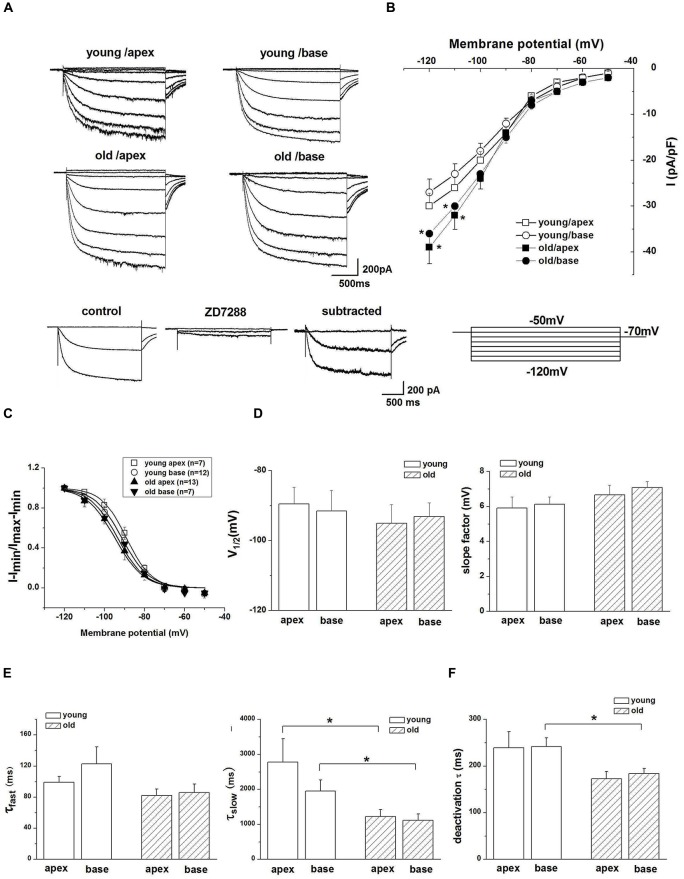
Ih recorded from SGNs of young and old mice. **(A)** Upper panel shows representative traces from the apex and base of the cochlea in response to 200-ms voltage steps from a holding potential of −60 mV to potential between −120 mV and −50 mV in 10 mV increments. The tail currents were obtained at −70 mV. Bottom row shows representative traces from SGNs of apex from young mice before and after application of 100 μM ZD7288, respectively. The subtracted traces represent ZD7288-sensitive current. For clarity some of the traces were omitted in the illustration. **(B)** Current density-voltage curve obtained from SGNs of apex and base from young and old mice (young: *n* = 12, old: *n* = 11). **(C)** Activation curve of tail currents fitted with the Boltzmann equation. Summaries of V_1/2_ and slope factors are shown in **(D)**, (*n* = 8). **(E)** Activation kinetics of Ih. Current responses to −120 mV voltage steps were measured by fitting a double exponential equation, providing two times constants (τ_fast_, τ_slow_; young apex: *n* = 9, old apex: *n* = 9, young base: *n* = 12, old base: *n* = 10). **(F)** Summary of mean deactivation time constants (*n* = 7). Data are means ± SEM, *p < 0.05.

We next attempted to identify the properties of HCN channels in SGNs and investigated whether upregulation of HCN channel in SGNs was related to aging. We recorded Ih, using whole-cell voltage-clamp technique in cultured SGNs. To verify that hyperpolarization-activated inward currents in SGNs were carried by HCN channels, we applied HCN channel blocker ZD7288 during recording. Figure 2A, bottom panel, shows Ih recorded in SGNs, which were isolated from the apex of the cochlea of young mice. The current was blocked completed by 100 μM ZD7288. A significant increase in current density of Ih was observed in SGNs from old mice compared with young ones (Figure 2B). To analyze the voltage dependence of Ih, activation curves were generated from tail currents (Figure 2C). There was no significant difference in half-activation voltage (V_1/2_) and slope factors of Ih between young and old SGNs (Figure 2D). Currents at both ages exhibited dual exponential time constants of activation, τ_fast_ and τ_slow_. While the fast component of the activation time constant, τ_fast_ did not show significant changes in SGNs from young vs. old mice (Figure 2E), the slow component, τ_slow_, was decreased for Ih in both apical and basal SGNs in old mice compared to young mice (Figure 2E, *p* < 0.05). In addition, the deactivation time constants of Ih showed a decrease in basal neurons in old SGNs (Figure 2F, *p* < 0.05). Together, these results showed that the upregulation of HCN channels resulted in an increase of Ih in SGNs from old mice.

### HCN Channels Contribute to Age-Related Changes of SGNs Membrane Properties

**Figure 3:**
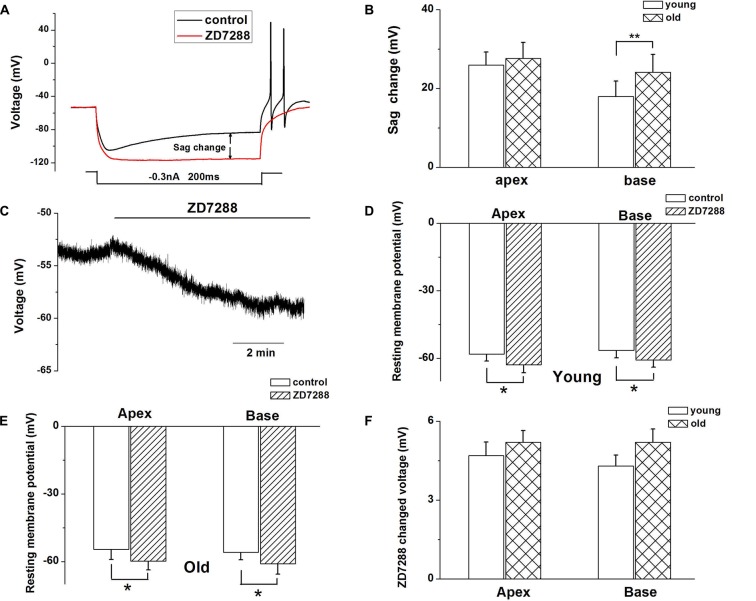
Ih contributes to SGN membrane responses. **(A)** Representative current-clamp recording from young basal SGN. Upon application of 100 μM ZD7288, the depolarization sag is abolished. **(B)** Quantification of sag amplitude before and after treatment of SGNs from young and old mice with ZD7288, *n* = 8. **(C)** Application of 100 μM ZD7288 induced membrane hyperpolarization in a basal SGN preparation. **(D,E)** Summary data of effect of ZD7288 on resting membrane potential (RMP; young: *n* = 12, old: *n* = 11). **(F)** shows changes in RMP in apical and basal SGNs of young and old mice (young: *n* = 12, old: *n* = 11). Data are means ± SEM, *p < 0.05, **p < 0.01.

Since HCN channels are activated at hyperpolarized membrane potentials and are important for regulating neuronal RMP, we examined whether upregulation of HCN channels and increased Ih contributed to the age-related changes in SGNs membrane properties. To characterize the effect of Ih on membrane potentials, we recorded the voltage sag in apical and basal SGNs from young and old mice in current-clamp configuration. In response to a −0.3-nA current injection for 200 ms, SGNs membrane voltage exhibited rapid hyperpolarization followed by a depolarizing voltage “sag” (Figure 3A). To confirm whether the sag resulted from Ih activation, we blocked Ih with ZD7288 and found that application of 100 μM ZD7288 abolished the sag (Figure 3A). We measured the voltage sag as the difference between the peak hyperpolarization and the steady-state potential at the end of the current step. The changes of sag amplitude induced by ZD7288 increased significantly in basal SGNs from old mice compared with young mice (*p* < 0.05). There were no significant changes in apical SGNs between young and old mice (Figure 3B). These results suggest that functional changes of HCN channels may be predominant in basal SGNs.

We then examined whether HCN channels contribute to the RMP (*V*_rest_) of SGNs during aging. ZD7288 produced a significant hyperpolarization of *V*_rest_ of SGNs (*p* < 0.05, Figures 3C–E). For young SGNs, application of ZD7288 hyperpolarized *V*_rest_ by 4.7 ± 0.5 mV at the cochlear apex and 4.3 ± 0.4 mV at the base of the cochlea (Figures 3D,F, Table 1). In contrast, in old mice SGNs, ZD7288 hyperpolarized *V*_rest_ by 5.2 ± 0.4 mV at the apex and 5.2 ± 0.5 mV at the base of the cochlea (Figures 3E,F, Table 1). However, the ZD7288-induced changes of *V*_rest_ were statistically unchanged in young SGNs compared with old ones (Figure 3F, Table 1).

**Table 1:** Effects of ZD7288 on membrane properties apical and basal spiral ganglion neurons (SGNs).

		Apex			Base		
	Age	*n*	Control	ZD7288	*n*	Control	ZD7288
Spike number	Young	12	1.0 ± 0	1.0 ± 0	14	17.4 ± 3.2	9.1 ± 1.1**
	Old	11	1.0 ± 0	1.0 ± 0	10	30.0 ± 4.7	6.3 ± 0.9**
Rmp (mV)	Young	12	−58.1 ± 3.1	−62.8 ± 3.6*	14	−56.4 ± 3.3	−60.7 ± 3.2*
	Old	11	−54.6 ± 4.5	−59.8 ± 3.9*	10	−55.8 ± 3.3	−61.0 ± 4.5*
AP duration (ms)	Young	12	3.8 ± 0.3	4.4 ± 0.3	14	3.3 ± 0.2	3.3 ± 0.3
	Old	11	3.6 ± 0.2	3.5 ± 0.3	10	4.0 ± 0.3	3.9 ± 0.4

*p < 0.05 ZD7288 vs. control. **p < 0.01.

### HCN Channels Contribute to the Changes of SGN Firing Properties

**Figure 4:**
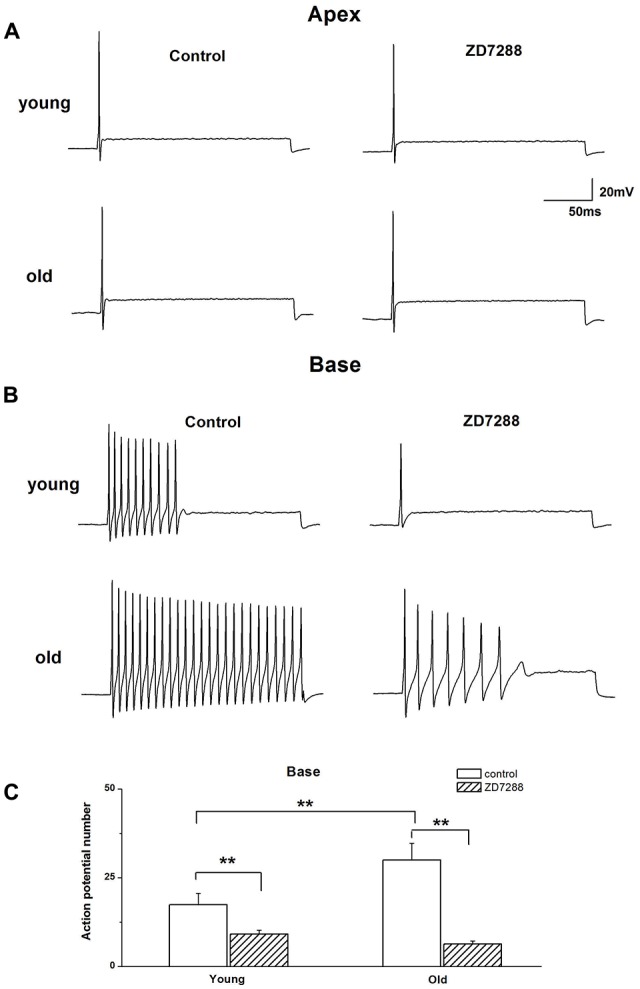
Effects of blockage of Ih on membrane excitability of SGNs. **(A)** Injection of 0.2 nA current produced single spikes in apical SGNs from young and old mice. **(B)** In contrast, injection of 0.2 nA produced multiple spikes in basal SGNs. **(C)** Summary data of the effect of ZD7288 on spike number in basal SGNs from young and old mice (young: *n* = 12, old: *n* = 11). Data are means ± SEM, **p < 0.01.

The preceding data showed the involvement of Ih in voltage “sag” changes and *V*_rest_ of SGNs in old mice. We examined whether Ih contributes to the age-related changes of action potentials (APs) in SGNs. We found there was an increase in AP numbers in basal SGNs in old mice compared with those in young neurons (young: 17.4 ± 3.2, *n* = 12; old: 30.0 ± 4.7, *n* = 11, *p* < 0.05). There was no significant difference in the response properties of apical SGNs between young and old mice. We applied 100 μM ZD7288 to investigate whether Ih contributes to these excitability changes. Suppression of Ih with ZD7288 dramatically reduced SGN firings in basal SGNs in young vs. old mice (47.7% decreased in young and 79.0% decreased in old mice, Figure 4C). The inhibitory effects on basal SGNs in old mice were more pronounced than in young mice (Figure 4B). By contrast, in apical SGNs, the AP profile and frequency of firing were unchanged between young and old SGNs (Figure 4A and Table 1).

### Loss of SGNs in Old Mice

**Figure 5:**
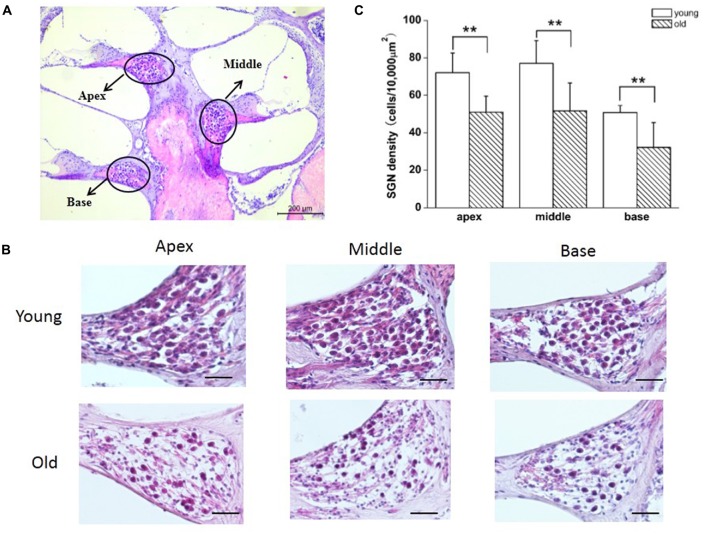
Old mice displayed degeneration of SGNs. **(A)** Cross section of complete cochlea showing all regions: apex, middle and base. **(B)** Morphometry changes of SGNs were observed in the apical, middle and basal regions of cochlea from young and old mice. **(C)** SGNs number were counted at all regions of cochlea from young and old mice (*n* = 6). Scale bar 200 μm in **(A)** and 50 μm in **(B)**. **Significantly different from young mice (*p* < 0.01).

Previous reports have shown that loss of SGNs contributed to AHL (Ryals and Westbrook, 1988; White et al., 2000; Linthicum and Fayad, 2009). We performed histological analysis on cochlear tissue sections from young and old mice. In agreement with these studies, all three regions of the cochlea, including apex, middle, and base, from young mice displayed no loss of SGNs, while in old mice, those regions displayed severe loss of SGNs (Figures 5A,B). To further confirm the histological results, we counted the number of SGNs, and found that SGN densities were decreased significantly in old, compared with young mice (Figure 5C).

### Increased HCN Channels and Ih May Contribute to SGNs Apoptosis in Old Mice

**Figure 6:**
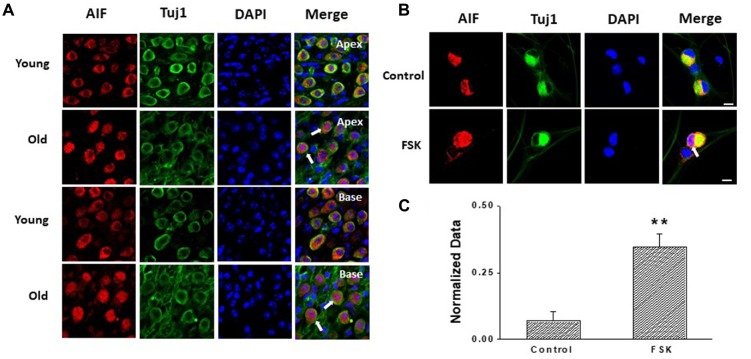
HCN channels contribute to apoptosis-inducing factor (AIF) translocation in SGNs. **(A)** AIF (red) was distributed in the cytoplasm of SGNs without overlap of nuclei (blue) in young mice. However, AIF overlapped with nuclei of SGNs (white arrow) in old mice which demonstrated that AIF translocated into nucleus. **(B)** Application of the HCN channel activator forskolin (FSK; 20 μM) for 24 h induced AIF translocation (white arrow) from the cytoplasm to the nucleus in cultured SGNs. AIF was stained red and nuclei were stained with DAPI (blue). SGNs were labeled with the neuronal marker Tuj1 (green). **(C)** Qualitative assessment of AIF translocation in cultured SGNs. The ratio of AIF translocated-positive SGNs to total SGNs was measured in control and FSK-treated group. AIF translocation and total SGNs were counted within five randomly low-power fields in three independent experiments. Scale bar: 10 μm. **p < 0.01.

Apoptosis is a major contributor to SGNs degeneration in the AHL, and HCN channels have been reported to participate in AIF-induced apoptosis in primary cultures of cortical neurons (Norberg et al., 2010). Accordingly, we predicted that changes in HCN channels would contribute towards SGNs apoptosis during AHL. We examined expression of apoptosis related protein in SGNs of old mice. We did not detect cleaved caspase-3 in old SGNs (Supplementary Figure S1), suggesting that other apoptotic pathways may be involved in this process. We thus investigated AIF-translocation in SGNs from young and old mice to explore whether AIF contributes to SGNs apoptosis. AIF is a flavor protein which during apoptosis is initially localized in mitochondria and migrates to the nucleus when caspase-independent apoptosis is involved. We determined potential correlates between increased expression and activity of HCN channels in AIF nuclear translocation. Our data showed that AIF was located mainly in the cytoplasm both in apical and basal SGNs in young mice. In old mice there was increased translocation of AIF from the cytoplasm to the nucleus (Figure 6A). To confirm whether HCN channels contribute to AIF translocation, we measured the effect of the adenylate cyclase agonist FSK, which can activate HCN channels in cultured SGNs, and found that 20 μM FSK increased AIF migration to the nucleus (Figures 6B,C). These results suggest that HCN channels may induce SGNs apoptosis through the AIF signaling pathway in the aging SGNs.

## Discussion

HCN channels play a key role in regulating neuronal functions (Ludwig et al., 1998; Robinson and Siegelbaum, 2003), such as determination of the RMP, generation of neuronal rhythmic oscillation, and regulation of dendritic integration and synaptic transmission. We report that in primary auditory neurons, also called SGNs, HCN channels and specifically, HCN1 and HCN2 channels, expression increased in young vs. old neurons. The ensuing increase in Ih mediates enhanced excitability of SGNs. Additionally, there is stark correlation between activation of Ih and AIF nuclear translocation. We propose that these age-dependent changes in SGN excitability and apoptotic related factor activation may be contributory factors involved in AHL.

### Increased Expression of HCN Channels in AHL

HCN channels form heterotetramers or homotetramers between HCN1–4 subunits *in vivo* and *in vitro*, and evaluation of HCN subunits expression in SGNs from young and old mice is critical for understanding their function. The expression pattern of HCN channels in SGNs may vary between different species and may undergo age-dependent changes. For example, Yi et al. (2010) found that HCN1, HCN2 and HCN4 proteins were expressed in neonatal rat SGNs. In another study, HCN1, HCN2 and HCN4 mRNA was detected in the mouse SGNs at both neonatal and postnatal stages (Kim and Holt, 2013). Moreover, Bakondi et al. (2009) reported that all four HCN subunits were expressed in adult guinea pig SGNs. In this study, we found that HCN subunits, including HCN1, 2, 3, 4, were expressed in SGNs from both young and old mice. The expression of HCN1 and HCN2 was significantly enhanced in SGNs from old mice, raising the possibility that upregulation of HCN channels may be involved in the mechanisms of age-dependent changes, membrane excitability and potential neurodegeneration associated with AHL.

### HCN Channels Contribute Towards SGN Excitability Changes in Old Mice

HCN channels consist of four subunits, and the presence and relative abundance of each subunit in each channel influences both the voltage dependence of the channel as well as its kinetics (Santoro et al., 2000). In the current study, increased expression of HCN1 and HCN2 subunits, was observed in SGNs from old mice, consistent with the prediction that upregulation of HCN may underlie mechanisms of alterations of SGNs excitability in old mice. We first recorded Ih in SGNs to observe whether Ih was increased in old mice. We report upregulation of HCN channel expression. The ensuing current, Ih is a slowly activating, non-inactivating inward current, and has been identified and characterized in SGNs of neonatal and young-age animals (Chen, 1997; Mo and Davis, 1997; Yi et al., 2010; Kim and Holt, 2013). However, the properties of Ih in SGNs from old mice were unknown. We found Ih was evoked by hyperpolarizing voltage steps with *V*_1/2_ = −94 ± 6 mV, *k* = 6.6 ± 0.5 mV in the apical SGNs, and *V*_1/2_ = −93 ± 4 mV, *k* = 7.1 ± 0.8 mV in basal SGNs. Meanwhile, the activation kinetics in SGNs from young mice was similar to those reported in previous studies (Chen, 1997; Kim and Holt, 2013). Furthermore, we found a dramatic increase in Ih density in older SGNs. However, the biophysical parameters of HCN channel currents did not show significant differences in young and old SGNs. Thus, increased Ih density may result from enhanced HCN channel protein expression rather than alterations of channel gating properties.

We explored how HCN channels regulated SGNs excitability in old mice. SGNs are situated around the axis of the cochlea, and show different morphological and electrophysiological specializations according to their cochlea location (Mo and Davis, 1997; Adamson et al., 2002; Lv et al., 2010, 2012). The basal region of cochlea transduces high frequency sound, while the apex transduces low frequency sound. Previous study in SGNs electrophysiological properties demonstrated a base-to-apex gradient in action potential number, duration, latency, RMP and potassium current densities (Lv et al., 2012). Therefore, we recorded HCN channel properties in SGNs from apical and basal regions of the cochlea. We found the AP number increased in basal SGNs from old mice compared with those from young ones, suggesting increased basal neuron excitability. The HCN channel blocker, ZD7288 dramatically reduced the APs number in basal SGNs from old mice, which strongly supports the inference that HCN channels in basal SGNs may play a critical role in the observed age-dependent changes.

### Upregulation of HCN Channels May Contribute Towards SGNs Apoptosis

SGNs loss along basal to apical contour of the cochlear axis in old mice have been reported previously and may contribute towards AHL. Apoptosis is one of the main causes of SGNs loss, which plays a key role in AHL (Jókay et al., 1998; Someya et al., 2007, 2009; Frisina et al., 2016). Activated caspase-8 and caspase-3 expression has been shown in SGNs from CBA/CaJ AHL mice, which suggests that caspase-dependent apoptosis may contribute to age-related SGNs loss (Frisina et al., 2016). However, another study reported that loss of SGNs in young adult mice lacked caspase-3, which indicates that activated caspase-3 is not essential for the death of SGNs (Takahashi et al., 2001). Additionally, Bak-mediated mitochondrial apoptosis of SGNs were observed in C57 AHL mice (Someya et al., 2009). These studies indicate that several apoptosis-related pathways may contribute to SGNs loss in AHL. In the current study, we did not detect cleaved caspase-3 in SGNs from old mice (Supplementary Figure S1). These findings support the conclusion that the caspase pathway may not be a major pathway for SGNs loss in C57 mice in HCN-upregulated-mediated mechanisms. AIF, Another important apoptosis-related protein, was observed to be translocated into the nucleus of SGNs in old mice, suggesting that AIF may mediate SGN death in C57 mice. AIF is a flavor protein, involved in redox reactions in the electron transport chain and induces apoptosis under conditions of injury (Otera et al., 2005; Hangen et al., 2010). It has been shown that AIF contributes to SGNs apoptosis during glutamate or peroxynitrite-induced injury (Liu W. et al., 2012; Ding et al., 2015). Our study suggests AIF-mediated apoptosis may be involved in SGNs loss in AHL. Norberg et al. (2010) demonstrated that HCN2 participated in the AIF-induced apoptosis pathway in primary cultures of cortical neurons by a Ca^2+^-dependent mechanisms. To further explore whether HCNs contribute to AIF-dependent apoptosis pathway, we applied FSK to activate HCN channels in cultured SGNs. We found that FSK caused AIF translocation from the cytoplasm to nucleus in SGNs, strongly suggesting that HCN channels may contribute to the AIF-induced apoptotic pathway. Thus, we identified an additional HCN-mediated AIF-related apoptosis in SGNs that may contribute to AHL in current study, consistent with previous reports (Someya et al., 2009).

## Conclusion

In summary, we found an upregulation of HCN channels in old SGNs mediates changes in SGNs excitability during AHL. Moreover, AIF translocation to the nucleus in SGNs from old mice is associated with loss of SGNs, which could be activated by HCN channels. Taken together, our results demonstrate that there is possible correlation between age-dependent increased expression of HCN channels and Ih, and apoptosis in SGNs, which may contribute towards one of the varied mechanisms of AHL.

## Author Contributions

PLv designed the research, analyzed data and wrote the article. HS designed and performed the research. WL performed the research and analyzed the data. QG, HL, ML, PLi and BZ performed the research. EY wrote the article and conceived the experiments.

## Conflict of Interest Statement

The authors declare that the research was conducted in the absence of any commercial or financial relationships that could be construed as a potential conflict of interest.

## Supplementary Material

FIGURE S1 Cleaved caspase-3 was not detected in SGNs from apex **(A)** and base **(B)** of young and old mice. SGNs were labeled with the neuronal marker Tuj1 (green), cleaved caspase-3 were stained red, and nuclei were stained with DAPI (blue). Scale bar 20 μm.
