# Supplementary figures and images for: Age-Dependent Up-Regulation of HCN Channels in Spiral Ganglion Neurons Coincide With Hearing Loss in Mice
Source: Front Aging Neurosci. 2018 Nov 6;10:353. doi: 10.3389/fnagi.2018.00353 (PMC6232381; doi:10.3389/fnagi.2018.00353)

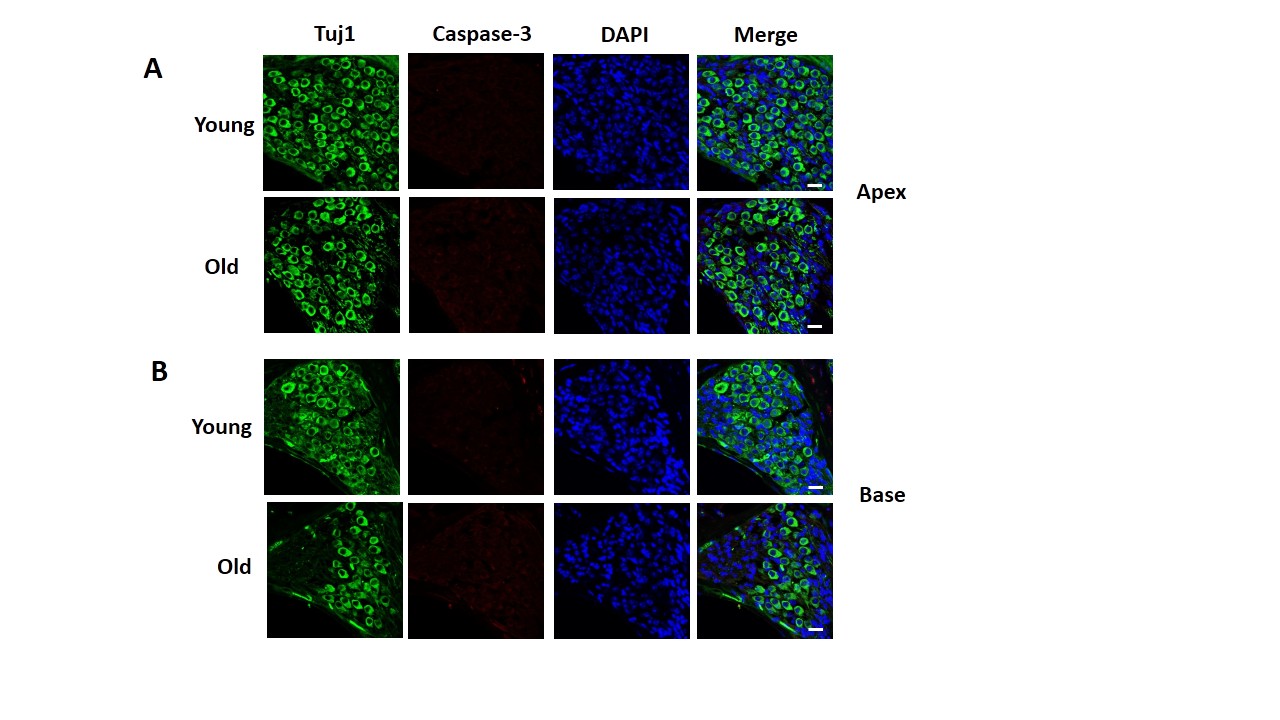

Supplement: FIGURE S1 — Cleaved caspase-3 was not detected in SGNs from apex (A) and base (B) of young and old mice. SGNs were labeled with the neuronal marker Tuj1 (green), cleaved caspase-3 were stained red, and nuclei were stained with DAPI (blue). Scale bar 20 μm. [file Image_1.JPEG]
